# COVID-19 related stigma, empathy and intention for testing in Jordan

**DOI:** 10.1371/journal.pone.0274323

**Published:** 2022-09-12

**Authors:** Ghada Shahrour, Latefa Dardas, Mohammed Aldalaykeh

**Affiliations:** 1 Department of Community and Mental Health Nursing, Faculty of Nursing, Jordan University of Science and Technology, Irbid, Jordan; 2 Department of Community and Mental Health Nursing, School of Nursing, The University of Jordan, Amman, Jordan; Kyung Hee University School of Medicine, REPUBLIC OF KOREA

## Abstract

Stigmatization of COVID-19 disease has been speculated due to misinformation about the disease, fearing of contracting the infection, absence of available cure, and holding responsibility for infecting others. We aimed to establish the prevalence of COVID-19 related stigma and its association with empathic responding, in addition to exploring predictors of stigma and testing intention among Jordanian people. A quantitative, descriptive and predictive design was used and data were collected using a web-based survey from 1074 adults. Findings showed that participants had high stigmatization against COVID-19 infection. Higher empathic responding (both cognitive and affective), being a female participant, and older age resulted in higher stigmatization. Only stigmatization of COVID-19 negatively predicted individuals’ intention for testing. These findings warrant intensive efforts from the Jordanian government on a local and national level to provide ongoing public education related to several aspects of COVID-19 disease, in order to reduce or prevent the associated stigma and increase people’s intention for testing.

## Introduction

The 2019 novel coronavirus (COVID-19) has emerged as a worldwide pandemic. The rapidness and aggressiveness of the coronavirus in inflicting people made it a serious and threatening global health issue. The COVID-19 by far has infected millions of people, resulting in thousands of deaths worldwide [1]. Many aspects related to COVID-19 led to stigmatization of the persons infected with the disease [2].

COVID-19 related stigma refers to a negative attitude towards those being infected or having close contact with COVID-19 cases [3]. Sources of stigmatization include: misinformation about the disease, fearing of contracting the infection, absence of available cure, and holding responsibility for infecting others [4]. Such stigmatization can be manifested in humor-prone stigma which creates an atmosphere of hatred and emotional protest against persons infected with COVID-19. Organizational, residential, and apathetic stigma can be surfaced as well. Organizational stigma is represented through refusing to treat COVID-19 suspected or infected cases and imposing individuals to self-quarantine, while residential stigma refers to forcing those suspected or infected people to leave their homes. On the other hand, apathetic stigma relates to lack of empathy towards family members, friends, or relatives once they are infected with the illness [4]. Recent studies reported that persons discharged from quarantine, individuals infected or suspected of having COVID-19, and people returning from oversees experienced some form of stigmatization, where they were socially excluded, insulted, and stereotyped [5]. In a study of households with at least one confirmed case of COVID-19, stigmatization resulted in the reluctance of those families to disclose their coronavirus status to others and to meet people after quarantine and isolation. They even avoided discussing their worries and fears of COVID-19 to their family members [6]. Some survivors of COVID-19 even reported being rejected by their neighbors, employers and landlords [7]. In a study comparing levels of stigma among COVID-19 survivors and healthy controls in China, survivors reported more overall stigma, resulting in social isolation and feelings of shame [8]. Another study from Vietnam, found that 34% of healthcare workers felt stigmatized and in return, avoided contacts with neighbors and others in the community, and 10% felt blameworthy by friends and relatives [9].

COVID-19 Stigmatization may lead to harassment, bullying, discrimination, and loss of social bonds and relationships. Health risks and psychological problems including depression and suicide were also reported [4, 10]. About 24.3% of a combined sample of participants from four low to middle income countries reported significant depressive symptoms (i.e., 11.1% for Togo, 30.8% Haiti, 27% RDC, and 20.6% Rwanda) due to stigma associated with the pandemic [11]. In Nigeria, COVID-19 stigmatization enacted by friends, colleagues, and residential communities against frontline healthcare workers resulted in the experience of emotional trauma and other psychological concerns among this group [12]. Stigma associated with COVID-19 may also cripple strategies aimed to control and prevent the spread of the pandemic as stigmatized people are less likely to disclose their health status [5], thus avoiding professional help-seeking behaviors [13], and refusing to take COVID-19 test [2]. Understanding whether stigma may impact COVID-19 testing is vital for identifying potential barriers for public health efforts to increase testing and thus, contain the disease.

News media played a crucial role in the development and intensification of stigma against health risks through intensifying the feelings of threat and fear against certain groups that were identified with the spread of the disease [14, 15]. For example, Canadian news during SARS outbreak contributed to the prejudice enacted against Chinese Canadians where fears and anxieties were directed against them, merely for associating this group with the origin of the outbreak in Hong Kong [16]. Social media can magnify the stigma associated with certain risk groups as news stories are shared thousands of times [17]. Moral panic, identified as periods of intense concern about the behavior of a group that poses threat to the safety of other was witnessed in COVID-19 pandemic. In Canada, individuals who were infected with COVID-19 were blamed for not following preventative measures. Those individuals were labelled “covidiots”, “irresponsible” “embarrassing” and careless [14]. In analyzing COVID-19-related tweets posted between December 31, 2019, and March 13, 2020, approximately 25% of the tweets had stigma-related content [18].

One factor that was found to reduce the effect of stigmatization is empathy. Empathy refers to the individual’s ability to understand and engage in another’s emotional state [19]. There is a consensus in the literature that empathy is composed of two components, cognitive and affective. Cognitive empathy (CE) refers to the ability of taking the perspective of others and understanding their emotional state; while the affective component deals with the emotional response that is congruent with the other’s emotional state [19]. Research has shown that empathy correlates negatively with stigma [20, 21]. The more empathy levels in terms of perspective taking and affective responding, the less stigma people may display [21]. High levels of empathy are associated with caring and supportive interactions with others. In the case of infectious diseases, empathy responding was associated with less avoidance of individuals who were perceived as being at high risk for contracting the infection. Individuals with high empathic responding were also found to engage in effective precautionary health behaviors to avoid contracting the disease and minimize the spread of infection to others [22, 23].

Jordan, similar to other countries in the world, has been suffering from increased number of COVID-19 cases which led the government to enforce several curfews beginning on March 21^st^, 2020. The intensive efforts of the Jordanian government to contain the disease through implementing strict safety measures such as long and short periods of curfew, active testing for suspected cases, closing schools and colleges and moving to home-based distance learning, and institutionalization of infected individuals raised the hopes of Jordanian population to have COVID-19 free country. Such hopes and strict measures led to stigmatization of COVID-19 infected cases. The huge use of social media and the close social network among Jordanian people, led to the publicity of the names of infected individuals who were blamed and stigmatized for contracting the disease. In one study, 64% of Jordanians showed stigmatization attitudes towards infected people and their contacts [24]. Such stigmatization led to bullying behaviors against infected cases and their associates. Jordanians believed that COVID-19 patients were highly bullied and a high percentage of the public enjoyed sharing patients’ identities or news on social media platforms [25]. Bullying behaviors were further extended to those who were associated with the origin of the disease. East and Southeast Asian students in one of the Jordanian public universities reported being bullied and stigmatized as a result of associating their nationality with the origin and spread of COVID-19 [26]. The scope of the aforementioned studies is limited in exploring the impact of COVID-19 stigmatization on Jordanians’ intention for testing and the role of individual’s empathy on stigma mitigation. Furthermore, international literature focused mainly on identifying rates of COVID-19 stigma and associated mental health consequences. The extent of stigmatization in those studies differed based on the county and population investigated. Mild stigma was reported among US citizens (i.e., 11.2% - 4.5%) [10], while COVID-19 patients in China indicated moderate degree of stigmatization [27]. On the other hand, healthcare workers providing care to dialysis patients [28] and patients infected with COVID-19 [29] encountered high levels of perceived stigma. According to Stangl and colleagues [30], stigma is considered context-specific which requires understanding stigma in the given context. Therefore, the aim of this study was to explore the prevalence of COVID-19 stigma and its impact on testing intention among Jordanian people. It also aimed to investigate the relationship between individual’s empathy and COVID-19 stigmatization. More specifically, this study was guided by the following research questions:

1. What is the prevalence of COVID-19 stigma among the Jordanian public?
2. What is the relationship between COVID-19 related stigma and empathy?
3. What are the predictors of COVID-19 related stigma among Jordanian people?
4. What are the predictors of COVID-19 testing intention among Jordanian individuals?

## Methodology

### Design

A quantitative, cross-sectional, descriptive and comparative design was used in this study to (a) explore the prevalence of COVID-19 related stigma, (b) investigate the correlation between COVID-19 related stigma and empathy, (c) examine predictors of COVID-19 related stigma, and (d) examine predictors of COVID-19 intention for testing among Jordanian adults.

### Sample and sample size

A convenience sampling procedure was used in this study. A total of 1074 Jordanian adults whose age is 18 years and above was recruited electronically. The survey was created using Google Forms which is an electronic survey, or more specifically a web-based survey to distribute the study questionnaire.

For sample size calculation, Daniel’s [31] formula was used to estimate the prevalence of COVID-19-related stigma. The following parameters were used: (a) a precision rate of 3%, (b) a 95% confidence interval (CI), and an average prevalence of 64% based on stigma prevalence of COVID-19 among Jordanian people [24]. Based on these parameters, the estimated sample size was 984 subjects.

### Data collection procedure

The survey link was distributed through several social media websites along with an invitation letter clarifying all aspects of the study including: the voluntary nature of the study, confidentiality of information, its purpose, and elicited benefits and risks. An online consent form was also developed and subjects were asked to read the consent form carefully and click on the agree button if they were willing to participate in the study. To ensure the anonymity of participants, the survey did not include any information that may lead to identify the identity of potential subjects. Nor there was a possibility of identifying such information once the survey was completed. Completed questionnaires were automatically saved on Google Forms which is password protected and only accessible by the study authors. The study was approved by the Institutional Review Board (IRB) of Jordan University of Science and Technology, (# 325–2020). Data collection started by July 2020 and ended by September 2020, yielding a total of 1074 responses.

### Measures

1. Sociodemographic Data Sheet: participants were asked to complete a sociodemographic data sheet containing participant’ age, gender, marital status, educational level, and income.
2. COVID-19-related stigma. This measure is an adaptation of the Stigmatization attitudes towards people living with HIV (SAT-PLWHA-S) [32] that assesses attitudes of stigma against individuals with HIV. Items were adapted to reflect the case of COVID-19 and items that read "HIV individuals" in the original measure was changed to "COVID-19 patients". The instrument’s items represent several subscales including: a) concerns about occasional encounters (e.g., being around someone who has COVID-19 does not bother me); fear of personal contact (e.g., I can not be friends with someone who has COVID-19; c) responsibility and blame (e.g., people infected with COVID-19 have only themselves to blame); d) liberalism (e.g., the spread of COVID-19 is linked to the decline of moral commitment to safety measures); e) discriminatory behaviors (e.g., if I had a roommate and discovered he/she was infected with COVID-19 virus, it would not bother me); f) confidentiality issues (I have to know if someone around me is infected with COVID-19 virus), and g) criminalization of transmission (e.g., transmitting COVID-19 virus is a crime). The scale is scored on a 4-point Likert scale ranging from 1 (strongly disagree) to 4 (strongly agree). The scale has shown adequate psychometric properties [32].
3. Interpersonal Reactivity Index [33]. This scale was used to measure individual’s cognitive and affective empathy. Only the two subscales of this measure will be used; perspective taking and emotional concern as they apply to the study’s objectives. Each subscale is composed of 7-items answered on a 5-point Likert scale, ranging from “Doesn’t describe me well” to “Describes me very well”. Reliabilities of these subscales were reported widely above .70 [34].
4. Intention for testing. Participants were asked to indicate their intention to take up COVID-19 testing on a 5-point Likert scale from 1 = very unlikely to 5 = very likely. This item is an adaptation of the intention for HIV-testing item from Mo, Lou, and Fong [35].

The aforementioned instruments were translated using the procedures of Brisling [36] and Chapman and Carter [37] for instrument translation. To ensure the reliability, validity, and cultural sensitivity of the instruments, a bilingual professional editor translated the measures form English to Arabic. After that, another bilingual professional editor back-translated the instruments. The translation was accomplished with three main purposes: (a) having conceptual equivalence of the original measures, (b) having clear and simple language; and (c) no jargon present. Furthermore, a panel of three experts in the area of instrument development and validation were consulted. These experts reviewed the translated measures and reached a consensus on the final version. Pilot-testing of the survey was conducted among 20 individuals who verified the final version of it.

### Analysis plan

The Statistical Package for Social Sciences version 24 was used for data entry and analysis. Descriptive statistics of frequencies, means, range, and standard deviations were calculated to describe participants’ demographics and empathy, in addition to the prevalence of Covid-19 related stigma. Pearson correlation was used to detect the relationship between COVID-19-related stigma and empathy. Pearson correlation is used to assess the linear relationship between two continuous variables [38] and in this research COVID-19 stigma and empathy were both continuous variables. Hierarchal multiple regression was used to test whether participants’ level of empathy could predict their stigma level above and beyond their gender, age, marital status, income, and education. Hierarchal multiple regression was also used to predict participants’ intention for COVID-19 testing, where sociodemographics and stigma subscales were entered as predictors. Multiple regression is used when the dependent variable in the regression analysis is continuous and the multiple independent variables are either continuous or categorical [38]. In this study, the dependent variable of each regression analysis (i.e., stigma in the first multiple regression analysis and intention for testing in the second multiple regression analysis) were continuous and the independent variables were either continuous or categorical.

## Results

### Sample characteristics

A total of 1074 Jordanian adults (60.4% females) completed and returned the online survey. The age group of 18–25 years constituted 39.7% of the total sample, followed by the age group of 26–33 (20.1%). About 53.4% of participants were single and 56.1% had a BSN degree. Regarding monthly income, 29.4% had a monthly income of between JD 300 to 500 (USD 423 to 705) and 29.1% had a monthly income less than JD 300 (less than USD 423) (See Table 1).

**Table 1:** Participants’ demographic characteristics (N = 1074).

Characteristic	Subgroups	*n*	*%*
Gender	Female	649	60.4%
	Male	425	39.6%
Age	18–25 years	426	39.7%
	26–33 years	216	20.1%
	34–40 years	201	18.7%
	41–50 years	138	12.9%
	51–60 years	77	7.2%
	Above 60 years	16	1.4%
Marital status	Single	574	53.4%
	Married	478	44.5%
	Divorced	16	1.5%
	Widow	6	.6%
Education	Less than high school	49	4.6%
	High school	55	5.1%
	Diploma	78	7.3%
	Bachelor	698	65.1%
	Master or higher	194	18%
Family monthly income	Less than JD300 ($423)	312	29.1%
	JD301-500 ($424–705)	316	29.4%
	JD501-700 ($707–988)	156	14.5%
	JD701-1000 (989–1411)	139	12.9%
	JD1001-1500 ($1413–2117)	78	7.3%
	> 1500 ($2117)	73	6.8%

### COVID-19 related stigma and empathy

The mean score of COVID-19 related stigma was 60.9 (SD = 7.84), ranging from 6–87. The mean scores of the stigma subscales ranged from 4.95 to 13.59 with the subscales of liberalism (M = 13.59, SD = 1.97, range 4–16), and criminalization of transmission (M = 6.76, SD = 1.19, range 2–8) had the highest scores; while concerns about occasional encounter had the lowest score (M = 4.95, SD = 2.07, range 1–12).

Using the Interpersonal Reactivity Index, the mean score of total empathy was 46.24 (SD = 5.77), ranging from 4–65. For cognitive empathy, the mean score was 24.51 (SD = 3.79), ranging from 1–35 and that for affective empathy was 21.73 (SD = 3.07), ranging from 1–32. Pearson correlation revealed a significant positive correlation between COVID-19 related stigma and empathy total score (*r* = .20, *p* < .001). Further analysis showed a significant positive relationship between stigma total score and cognitive empathy (*r* = .18, *p* < .001) and affective empathy (*r* = .16, *p* < .001). Results related to stigma and empathy total and subscales are described in Table 2.

**Table 2:** Descriptive statistics of stigma and empathy total and subscales (N = 1074).

	M (SD)	Median	Range
**Stigma Total**	60.9 (7.8)	61	6–87
Concerns about occasional encounters	4.9 (2.07)	4	1–12
Fear of personal contact	5.4 (2.2)	5	1–12
Responsibility/ blame	11.0 (3.0)	11	1–20
Liberalism	13.5 (1.9)	14	4–16
Discriminatory behaviors	10.9 (2.2)	11	1–16
Confidentiality of testing	8.4 (1.4)	9	3–12
Criminalization of transmission	6.7 (1.2)	7	2–8
**Empathy Total**	46.2 (5.7)	46	4–65
Cognitive empathy	24.5 (3.7)	25	1–35
Affective empathy	21.7 (3.0)	22	1–32

### Predictors of COVID-19 related stigma

The hierarchal multiple regression results showed that the first model (containing participants’ gender, age, marital status, educational level, and income) significantly predicted COVID-19 stigmatization (F (5, 1015) = 10.48, *p* < .001, R^2^ = .044). However, the second model explained more variability of stigma variance (F (7,1013) = 13.95, *p* < .001, R^2^ = 0.082). Approximately 8% of the variability in COVID-19 related stigma was accounted for by participants’ gender, age, and cognitive and affective empathy, while other predictors of marital status, income, and education were not significant. More specifically, female participants (β = −.08, t (1013) = −-2.80, *p* < .01), older age (β = .21, t (1013) = 5.43, *p* < .001), and higher cognitive and affective empathy, (β = .12, t (1013) = 3.86, *p* < .001) and (β = .10, t (1013) = 3.27, *p* = .001), respectively, resulted in higher COVID-19 related stigma. Table 3 shows the model fit.

**Table 3:** Predictors of COVID-19 related stigma (N = 1,074).

	Predictor	DF	SE	t Value	B	P Value
**Model 1**	Gender	5	.383	-2.63	-1.00	.008*
	Age	5	.179	5.40	.965	.000**
	Marital status	5	.411	-.708	-.291	.479
	Income	5	.138	-1.72	-.238	.085
	Education	5	.220	-2.29	-.504	.022*
	**R**^**2**^ **= .049**	**rR**^**2**^ **= .044**	**F = 10.48**			
**Model 2**	Gender	7	.377	-2.807	-1.058	.005**
	Age	7	.175	5.432	.951	.000***
	Marital status	7	.404	-.942	-.380	.346
	Income	7	.135	-1.794	-.243	.073
	Education	7	.216	-1.818	-.393	.069
	Cognitive empathy	7	.052	3.862	.202	.000***
	Affective empathy	7	.065	3.277	.213	.001**
	**R**^**2**^ **= .088**	**rR**^**2**^ **= .082**	**F = 13.95**			

DF: Level of Freedom; SE: Standard Error; B: Regression Coefficient

* *p* < .05

** *p* < .01

*** *p* < .001

### Predictors of intention for testing

The results of the hierarchal regression model revealed that for the first model (containing gender, age, marital status, educational level, and income) did not significantly predict participants’ intention for testing (F (5, 1018) = .816, *p* = .539, R^2^ = 0.004). However, the second model showed that only stigma subscales significantly predicted intention for COVID-19 testing (F (12, 1011) = 9.13, *p* < .001, R^2^ = 0.08). About 8% of the variability in participants’ intention for testing could be explained by participants’ stigmatization of COVID-19. More specifically, the subscales of concerns about occasional encounter (β = -.06, t (1011) = -1.99, *p* = .04), fear of personal contact (β = -.07, t (1011) = -2.17, *p* = .03), and discriminatory behaviors (β = -.08, t (1011) = -2.27, *p* = .02), negatively predicted intention for testing. On the other hand, liberalism (β = .17, t (1011) = 4.08, *p* < .001) and criminalization of transmission (β = .12, t(1011) = 3.53, *p* < .001) significantly and positively predicted intention of COVID-19 testing. Table 4 shows the model fit.

**Table 4:** Predictors of COVID-19 intention for testing (N = 1,074).

	Predictor	DF	SE	t Value	B	P Value
**Model 1**	Gender	5	.051	1.266	.064	.206
	Age	5	.024	.272	.006	.786
	Marital status	5	.055	. 690	.038	.490
	Income	5	.018	-.447	-.008	.655
	Education	5	.029	-1.180	-.034	.238
	**R**^**2**^ **= .004**	**rR**^**2**^ **= .001**		**F = .816**		
**Model 2**	Gender	12	.049	.740	.036	.459
	Age	12	.023	.409	.010	.682
	Marital status	12	.052	.638	.333	.524
	Income	12	.018	-.268	-.005	.788
	Education	12	.028	-1.436	-.040	.151
	Concerns about occasional encounter	12	.013	-1.997	-.025	.046*
	Fear of personal contact	12	.012	-2.173	-.026	.030*
	Responsibility/blame	12	.009	-1.082	-.009	.280
	Liberalism	12	.014	4.808	.069	.000***
	Discriminatory behaviors	12	.014	-2.271	-.033	.023*
	Confidentiality of testing	12	.018	1.887	.033	.059
	Criminalization of transmission	12	.023	3.539	.080	.000***
	**R**^**2**^ **= .098**	**rR**^**2**^ **= .087**		**F = 9.31**		

DF: Level of Freedom; SE: Standard Error; B: Regression Coefficient

* *p* < .05

** *p* < .01

*** *p* < .001

## Discussion

The findings of the current study revealed that Jordanian adults had high stigmatization against COVID-19 infection. This is also indicated by the high mean scores of the liberalism and criminalization of transmission subscales. More specifically, individuals viewed that the Jordanian community needs to have a strong commitment to adhere to the protective measures that are taken on personal and governmental level to protect against COVID-19 transmission. They also viewed individuals who are not adherent to COVID-19 preventative measures and transmit the infection to others as criminals. Interestingly, our findings also showed that the sample had high empathic abilities, both cognitive and affective and they were positively correlated with COVID-19 associated stigma. In addition to having cognitive and affective empathy as significant predictors of stigma, female participants and those of older age also predicted COVID-19 stigmatization.

In the current study, only stigmatization of COVID-19 predicted individuals’ intention for testing. Participants who fear personal contact and had concerns of being in contact with infected patients were more reluctant to undergo COVID-19 testing. Discriminatory behaviors also resulted in individuals being more deterrent in testing for COVID-19. On the other hand, liberalism or bearing values regarding COVID-19 preventative measures and criminalization of transmission of those who are not committed to the safety measures, positively affected participants’ intention for testing.

Social stigma associated with COVID-19 has been reported in the literature, with rates varying between mild to severe levels of stigmatization. For instance, mild stigmatization towards COVID-19 patients was reported among US citizens [10] and 25% of COVID-19-related tweets posted between December 31, 2019 and March 13, 2020, had stigma-related content [18]. On the other hand, patients infected with COVID-19 in China indicated moderate level of being stigmatized [27]. Our findings are in line with those studies reporting high rates of COVID-19-related stigma such as those conducted among dialysis staff [28] and healthcare workers providing care to COVID-19 patients [29]. High stigmatization attitudes were also reported among Jordanian individuals towards infected people and their contacts [24]. Stangl and colleagues [30] argued that stigma is context specific and thus, the status of COVID-19 pandemic during our data collection may explain the high levels of stigmatization concerning COVID-19. During that time, the curve of infected cases in Jordan was low compared with other countries and the country was under curfew/quarantine in an attempt to contain the disease transmission. Furthermore, the government imposed strict measures for containing the disease including wearing face mask and social distancing. Therefore, these imposed measures and the hopes of the Jordanians to have COVID-19 free country, led people to highly stereotype, harass, and bully those who are infected and their associates. Bullying behaviors were evident against East and Southeast Asian university students residing in Jordan during the pandemic due to the association of the origin and spread of the disease to their nationality [26]. Furthermore, a high percentage (i.e., 86.9%) of Jordanians believed that people in Jordan excessively bullied patients infected with COVID-19 [25]. Those reasons explain the high score of the liberalism subscale (which included items related to the implementation and adherence to COVID-19 protective measures) and criminalization of transmission subscale.

We would expect that high empathic responding would result in less social stigmatization. Understanding what others are feeling by adopting their perspective and responding in supportive ways (empathic responding), has been associated with less stigmatization of individuals with infectious and non-infectious diseases including HIV [39, 40] and mental disorders [20, 41], respectively. However, our data indicated the contrary; our participants who reported high empathic responding had higher COVID-19 social stigmatization and this may relate to the high contractibility and possible lethality of the disease, in addition to the uncertainties surrounding the disease nature. Furthermore, empathic responding in our study assessed trait empathy and did not target empathic responding towards individuals with suspected or infected cases of COVID-19. Current literature is lacking on the role of empathy on stigma in the context of COVID-19, therefore, future research needs to address the relationship between these two concepts.

Our study showed that female participants had higher COVID-19 social stigmatization than males. Gender differences in disease-related stigmatization have been reported in studies of HIV and mental illness, but not in the context of COVID-19. The literature inferred from those studies, showed that men rather than women had higher stigmatization [42–44]. For example, in the context of mental illness, women desired less social distance than men from individuals diagnosed with mental illness [42, 43] and had better attitudes towards individuals with HIV [44]. It is gender roles than gender per se that affects gender differences in stigma. Sandelowski and colleagues [45] explain that women tend to make a decision based on preserving social relations and moral identities, prioritizing others’ needs and welfare, and preventing harm to others. Therefore, it is not surprising that our female participants held higher social stigmatization of COVID-19 than men, given the threats this disease impose on the lives of female associates (i.e., her family). Women are found to take COVID-19 more seriously and follow safety measures better than men and this is due to women’s tendency to feel responsible about their family’s health [46]. Available studies showed that women experienced more fear and worry of being infected with COVID-19 and had higher risk perception of being infected compared to men [47, 48].

Our findings of the association between increased age resulting in higher COVID-19 stigmatization, is congruent with available research. Older people in the general population of China [49] and older healthcare workers [29] were more likely to endorse stigmatization attitudes to patients with COVID-19. Krendl and Wolford [50] explain that older adults tend to believe that individuals with undesirable condition holds responsible and accountable for their condition. Old age has also been reported as one of COVID-19 risk factors [2] which may explain the tendency to stereotype individuals infected with COVID-19, stemming from their fear of contracting the infection and dealing with its adverse outcomes.

The current study revealed that stigmatization of COVID-19 resulted in participants’ reluctance in undertaking COVID-19 testing. Similar findings were reported from a study conducted on a sample of United States adults where greater COVID-19 stereotypes led to participants’ unwillingness and hesitancy to seek a COVID-19 test [51]. More specifically, intention for testing in this study was negatively associated with fear of personal contact, if concerns of occasional encounter were not suspected, and if the participant lacked liberal views (i.e., had discriminatory behaviors). It seems that participants who feared personal contact and avoided occasional encounter of confirmed and previously infected individuals with COVID-19, have lower perception of risk, which justifies their unintentionality for testing. In their study of HIV intention for testing among the general population in Thailand, Musumari and colleagues [52] reported that the major reason for not testing was the perception of having no or low risk of being infected with HIV. Discriminatory behaviors as indicated by the negative views regarding the right of individuals who were infected with COVID-19 to resume their life after they get cured, was negatively correlated with intention for testing. Such strict attitudes implicate higher stigmatization towards individuals infected with COVID-19 and thus the fear of similar stigmatization if being in similar position, explains participants’ reluctance to undertake the testing. On the other hand, participants who held positive values (liberalism) regarding COVID-19 safety measures and viewed the transmission of infection as a criminal act, had higher intention for testing. Such attitudes may be explained by participants’ high perception of the seriousness and negative consequences the COVID-19 infection may inflict on their lives and those of their associates. In line with models of health believes and behaviors [53, 54], perceived threat of disease has been associated with taking health precautions in response to SARS [23] and West Nile [55] and vaccination uptake against H1N1 [56].

### Limitations

This study is considered the first to investigate COVID-19 related stigma in association with the public’s empathy and intention for testing, however, this study has some limitations. The data collection occurred at one-time point and relied on self-report measures of the study variables. Assessing the magnitude of social stigma related to COVID-19 among the public over time where changes in infection rate occur is needed. Furthermore, this study measured trait empathy and it would be of interest to see if differences in COVID-19 related stigma in relation to state empathy is found. Future research would also benefit from studying social stigmatization amid COVID-19 employing a variety of research designs (i.e., qualitative and mixed method design).

## Conclusion and implications

The current study aimed to investigate the prevalence of COVID-19 stigmatization and its relationship with individual’s empathy, in addition to explore predictors related to individual’s stigma and intention for COVID-19 testing among Jordanian people. The findings show that stigmatization against contracting COVID-19 is high among the Jordanian public. Several strategies can be taken by the government in Jordan to combat such stigmatization, especially stigma was shown to affect people’s testing intention and resulted in lower empathy towards COVID-19 patients. Educational anti-stigma interventions through presenting factual information and correcting misinformation or contradicting the prevailing negative attitudes and behaviors regarding COVID-19 is essential. Educational campaigns can be designed and targeted at any level from local to national to provide factual and up to date accurate information of the disease. Educational campaigns by the Jordanian government can be delivered either face to face by health professionals or via social media since the latter is considered a viable vector in intensifying public stigmatization of COVID-19.

## Supporting information

S1 Dataset (SAV)

## References

[1] Cennimo DJ. What is the global and US prevalence of coronavirus disease 2019 (COVID-19)? 2020. Available from https://www.medscape.com/answers/2500114-197405/what-is-the-global-and-us-prevalence-of-coronavirus-disease-2019-covid-19

[2] Turner-Musa J, Ajayi O, Kemp L. Examining social determinants of health, stigma, and covid-19 disparities. Healthcare. 2020;8(2):168. doi: 10.3390/healthcare8020168 32545647 PMC7349778

[3] Bhanot D, Singh T, Verma SK, Sharad S. Stigma and discrimination during COVID-19 pandemic. Front Public Health. 2021;8. doi: 10.3389/fpubh.2020.577018 33585379 PMC7874150

[4] Mahmud A, Islam MR. Social Stigma as a barrier to covid-19 responses to community well-being in Bangladesh. Int J Community Wellbeing. 2020;4(3):315–21. doi: 10.1007/s42413-020-00071-w 34723103 PMC7416994

[5] Adom D, Adu Mensah J. The psychological distress and mental health disorders from COVID-19 stigmatization in Ghana. Soc Sci Humanit Open. 2020. 10.1016/j.ssaho.2021.100186 PMC8257423 34250461

[6] Lohiniva A-L, Dub T, Hagberg L, Nohynek H. Learning about covid-19-related stigma, quarantine and isolation experiences in Finland. PLOS ONE. 2021;16(4). doi: 10.1371/journal.pone.0247962 33852581 PMC8046198

[7] Bu NN. The 80 thousand COVID-19 survivors are undergoing discrimination. 2020. Available from http://k.sina.com.cn/article_1690367810_64c0f74201900py8d.html?from=mood

[8] Yuan Y, Zhao Y-J, Zhang Q-E, Zhang L, Cheung T, Jackson T, et al. Covid-19-related stigma and its sociodemographic correlates: A comparative study. Global Health. 2021;17(1). doi: 10.1186/s12992-021-00705-4 33962651 PMC8103123

[9] Do Duy C, Nong VM, Ngo Van A, Doan Thu T, Do Thu N, Nguyen Quang T. covid ‐19‐related stigma and its association with Mental Health of health‐care workers after quarantine in Vietnam. Psychiatry Clin Neurosci. 2020;74(10):566–8. doi: 10.1111/pcn.13120 Epub 2020 Aug 25. ; PMCID: PMC7404653. 32779787 PMC7404653

[10] Pan SW, Shen GC, Liu C, Hsi JH. Coronavirus stigmatization and psychological distress among Asians in the United States. Ethn Health. 2020;26(1):110–25. doi: 10.1080/13557858.2020.1849570 Epub 2020 Dec 12. . 33307773

[11] Cénat JM, Noorishad P-G, Kokou-Kpolou CK, Dalexis RD, Hajizadeh S, Guerrier M, et al. Prevalence and correlates of depression during the COVID-19 pandemic and the major role of stigmatization in low- and middle-income countries: A multinational cross-sectional study. Psychiatry Res. 2021;297:113714. doi: 10.1016/j.psychres.2021.113714 Epub 2021 Jan 8. ; PMCID: PMC7837092. 33453497 PMC7837092

[12] Kwaghe AV, Kwaghe VG, Habib ZG, Kwaghe GV, Ilesanmi OS, Ekele B, et al. Stigmatization and psychological impact of covid-19 pandemic on Frontline Healthcare Workers in Nigeria: A qualitative study. BMC Psychiatry .2021;21(518). doi: 10.1186/s12888-021-03540-4 34670530 PMC8528377

[13] Duan W, Bu H, Chen Z. Covid-19-related stigma profiles and risk factors among people who are at high risk of contagion. Soc Sci Med. 2020;266:113425. doi: 10.1016/j.socscimed.2020.113425 33059301 PMC7540249

[14] Capurro G, Jardine CG, Tustin J, Driedger M. Moral panic about “covidiots” in Canadian newspaper coverage of covid-19. PLOS ONE. 2022;17(1). doi: 10.1371/journal.pone.0261942 35041667 PMC8765660

[15] Kasperson RE, Renn O, Slovic P, Brown HS, Emel J, Goble R, et al. The social amplification of risk: A conceptual framework. Risk Anal. 1988;8(2):177–87.

[16] Leslie M. Fear and coughing in Toronto: SARS and the uses of risk. Can J Commun. 2006;31(2): 367–389. 10.22230/cjc.2006v31n2a1544

[17] Ahmed W, Bath PA, Sbaffi L, Demartini G. Moral panic through the lens of Twitter. Proceedings of the 9th International Conference on Social Media and Society. New York, NY, USA: Association for Computing Machinery. 2018.

[18] Li Y, Twersky S, Ignace K, Zhao M, Purandare R, Bennett-Jones B, et al. Constructing and communicating COVID-19 stigma on Twitter: A content analysis of tweets during the early stage of the COVID-19 Outbreak. Int J Environ Res Public Health. 2020;17(18):6847. doi: 10.3390/ijerph17186847 32961702 PMC7557581

[19] Smith A. Cognitive empathy and emotional empathy in human behavior and evolution. Psychol Rec. 2006;56(1):3–21.

[20] McFarland S. Authoritarianism, social dominance, and other roots of generalized prejudice. Polit Psychol. 2010;31(3):453–77. doi: l0.1111/j.14679221.2010.00765.x

[21] Knolhof QA. The Role of Contact and Empathy in Stigma toward Individuals with Mental Illness among Mental Health and Non-Mental Health Professionals. M.Sc. Thesis, Eastern Illinois University. 2018. Available from https://thekeep.eiu.edu/cgi/viewcontent.cgi?article=4444&context=theses

[22] King DB, Kamble S, DeLongis A. Coping with influenza A/H1N1 in India: Empathy is associated with increased vaccination and health precautions. Int J Health Promot Educ. 2016;54(6):283–94. 10.1080/14635240.2016.1174950

[23] Lee-Baggley D, DeLongis A, Voorhoeave P, Greenglass E. Coping with the threat of severe acute respiratory syndrome: Role of threat appraisals and coping responses in health behaviors. Asian J Soc Psychol. 2004;7(1):9–23. doi: 10.1111/j.1467-839X.2004.00131.x 32313437 PMC7159516

[24] Abuhammad S, Alzoubi KH, Khabour O. Fear of Covid‐19 and stigmatization towards infected people among Jordanian people. Int J Clin Prac. 2020;75(4). doi: 10.1111/ijcp.13899 Epub 2020 Dec 18. ; PMCID: PMC7883188. 33280204 PMC7883188

[25] Akour A, AlMuhaissen SA, Nusair MB, Al-Tammemi AB, Mahmoud NN, Jalouqa S, et al. The untold story of the covid-19 pandemic: Perceptions and views towards social stigma and bullying in the shadow of covid-19 illness in Jordan. SN Soc Sci. 2021;1(9): 240. doi: 10.1007/s43545-021-00252-0 Epub 2021 Sep 27. ; PMCID: PMC8475478. 34693341 PMC8475478

[26] Alsawalqa RO. Cyberbullying, social stigma, and self-esteem: The impact of covid-19 on students from East and Southeast Asia at the University of Jordan. Heliyon. 2021;7(4): e06711. doi: 10.1016/j.heliyon.2021.e06711 33869877 PMC8045034

[27] Lin B, Zhong G, Liang Z, Huang J, Wang X, Lin Y. Perceived-stigma level of COVID-19 patients in China in the early stage of the epidemic: A cross-sectional research. PLOS ONE. 2021;16(10): e0258042. doi: 10.1371/journal.pone.0258042 34597354 PMC8486130

[28] Uvais NA, Aziz F, Hafeeq B. Covid-19-related stigma and perceived stress among dialysis staff. J Nephrol. 2020;33(6):1121–2. doi: 10.1007/s40620-020-00833-x 32804354 PMC7429935

[29] Nashwan AJ, Valdez GF, AL-Fayyadh S, Al-Najjar H, Elamir H, Barakat M, et al. Stigma towards health care providers taking care of COVID-19 patients: A multi-country study. Heliyon. 2022;8(4): e09300. doi: 10.1016/j.heliyon.2022.e09300 Epub 2022 Apr 18. ; PMCID: PMC9015722 35464709 PMC9015722

[30] Stangl AL, Earnshaw VA, Logie CH, van Brakel W, C. Simbayi L, Barré I, et al. The health stigma and discrimination framework: A global, crosscutting framework to inform research, Intervention Development, and policy on health-related stigmas. BMC Med. 2019;17(1). 10.1186/s12916-019-1271-3 PMC6376797 30764826

[31] Daniel WW. Biostatistics: A foundation for analysis in the Health Sciences. Hoboken, New York: Wiley; 1999.

[32] Beaulieu M, Adrien A, Potvin L, Dassa C. Stigmatizing attitudes towards people living with HIV/AIDS: Validation of a measurement scale. BMC Public Health. 2014;14(1). doi: 10.1186/1471-2458-14-1246 25476441 PMC4289343

[33] Davis MH. Measuring individual differences in empathy: Evidence for a multidimensional approach. J Pers Soc Psychol. 1983;44(1):113–26.

[34] Hawk ST, Keijsers L, Branje SJ, Graaff JV, Wied Mde, Meeus W. Examining the interpersonal reactivity index (IRI) among early and late adolescents and their mothers. J Pers Assess. 2012;95(1):96–106. doi: 10.1080/00223891.2012.696080 22731809

[35] Mo PK, Lau JT, Xin M, Fong VW. Understanding the barriers and factors to HIV testing intention of women engaging in compensated dating in Hong Kong: The application of the extended theory of planned behavior. PLOS ONE. 2019;14(6). 10.1371/journal.pone.0213920 PMC6597045 31246969

[36] Brislin RW. Back-translation for Cross-Cultural Research. J Cross Cult Psychol. 1970;1(3):185–216.

[37] Chapman DW, Carter JF. Translation procedures for the cross cultural use of measurement instruments. Educ Eval Policy Anal.1979;1(3):71–6. 10.3102/0162373700 1003071

[38] Lee SW. Regression analysis for continuous independent variables in medical research: Statistical standard and guideline of life cycle committee. Life Cycle. 2022;2:e3. 10.54724/lc.2022.e3

[39] Galinsky AD, Moskowitz GB. Perspective-taking: Decreasing stereotype expression, stereotype accessibility, and in-group favoritism. J Pers Soc Psychol. 2000;78(4):708–24. doi: 10.1037//0022-3514.78.4.708 10794375

[40] Olapegba PO. Empathy, knowledge, and personal distress as correlates of HIV-/AIDS-related stigmatization and discrimination. J Appl Soc Psychol. 2010;40(4):956–69.

[41] Corrigan PW, Rowan D, Green A, Lundin R, River P, Uphoff-Wasowski K, et al. Challenging two mental illness stigmas: Personal responsibility and dangerousness. Schizophr Bul. 2002;28(2):293–309. doi: 10.1093/oxfordjournals.schbul.a006939 12693435

[42] Phelan JE, Basow SA. College students’ attitudes toward mental illness: An examination of the stigma process. J Appl Soc Psychol. 2007;37(12):2877–902.

[43] Smith AL, Cashwell CS. Social distance and mental illness: Attitudes among mental health and non-mental health professionals and trainees. The Professional Counselor. 2011;1(1):13–20.

[44] Treves-Kagan S, El Ayadi AM, Pettifor A, MacPhail C, Twine R, Maman S, et al. Gender, HIV testing and stigma: The association of HIV testing behaviors and community-level and individual-level stigma in rural South Africa differ for men and women. AIDS Behav. 2017;21(9):2579–88. doi: 10.1007/s10461-016-1671-8 28058565 PMC5498263

[45] Sandelowski M, Lambe C, Barroso J. Stigma in HIV‐positive women. J Nurs Scholarsh. 2004;36(2):122–8. doi: 10.1111/j.1547-5069.2004.04024.x 15227758

[46] Healthline. Why Women Are Taking the COVID-19 Pandemic More Seriously Than Men. 2020. Available from https://www.healthline.com/health-news/why-women-are-taking-the-covid-19-pandemic-more-seriously-than-men

[47] Alnazly E, Khraisat OM, Al-Bashaireh AM, Bryant CL. Anxiety, depression, stress, fear and social support during COVID-19 pandemic among Jordanian Healthcare Workers. PLOS ONE. 2021;16(3). 10.1371/journal.pone.0247679 PMC7954309 33711026

[48] Wang C, Pan R, Wan X, Tan Y, Xu L, Ho CS, et al. Immediate psychological responses and associated factors during the initial stage of the 2019 coronavirus disease (covid-19) epidemic among the general population in China. Int J Environ Res Public Health. 2020;17(5):1729. doi: 10.3390/ijerph17051729 32155789 PMC7084952

[49] Zhang T-M, Fang Q, Yao H, Ran M-S. Public stigma of COVID-19 and its correlates in the general population of China. Int J Environ Res Public Health. 2021;18(21):11718. doi: 10.3390/ijerph182111718 34770234 PMC8582727

[50] Krendl AC. Reduced cognitive capacity impairs the malleability of older adults’ negative attitudes to stigmatized individuals. Exp Aging Res. 2018;44(4):271–83. doi: 10.1080/0361073X.2018.1475152 29781770

[51] Earnshaw VA, Brousseau NM, Hill EC, Kalichman SC, Eaton LA, Fox AB. Anticipated stigma, stereotypes, and covid-19 testing. Stigma Health. 2020;5(4):390–3. 10.1037/sah0000255

[52] Musumari PM, Techasrivichien T, Srithanaviboonchai K, Tangmunkongvorakul A, Ono-Kihara M, Kihara M. Factors associated with HIV testing and intention to test for HIV among the general population of Nonthaburi Province, Thailand. PLOS ONE. 2020;15(8). doi: 10.1371/journal.pone.0237393 32797048 PMC7428091

[53] Glanz K, Bishop DB. The role of Behavioral Science Theory in development and implementation of public health interventions. Annu Rev Public Health. 2010;31(1):399–418. doi: 10.1146/annurev.publhealth.012809.103604 20070207

[54] Rosenstock IM. The health belief model and Preventive Health Behavior. Health Educ Monogr. 1974;2(4):354–86. 10.1177/109019817800600406 299611

[55] Puterman E, Delongis A, Lee-Baggley D, Greenglass E. Coping and health behaviours in times of global health crises: Lessons from sars and West Nile. Glob Public Health. 2009;4(1):69–81. doi: 10.1080/17441690802063304 19153931

[56] Bish A, Yardley L, Nicoll A, Michie S. Factors associated with uptake of vaccination against pandemic influenza: A systematic review. Vaccine. 2011;29(38):6472–84. doi: 10.1016/j.vaccine.2011.06.107 21756960

